# Butyrylcholinesterase Protein Ends in the Pathogenesis of Alzheimer’s Disease—Could *BCHE* Genotyping Be Helpful in Alzheimer’s Therapy?

**DOI:** 10.3390/biom9100592

**Published:** 2019-10-09

**Authors:** Jacek Jasiecki, Bartosz Wasąg

**Affiliations:** 1 Faculty of Pharmacy with Subfaculty of Laboratory Medicine, Medical University of Gdańsk, 80-416 Gdańsk, Poland; 2 Department of Biology and Medical Genetics, Medical University of Gdańsk, 80-211 Gdańsk, Poland; bwasag@gumed.edu.pl; 3 Laboratory of Clinical Genetics, University Clinical Centre, 80-952 Gdańsk, Poland

**Keywords:** Alzheimer’s disease, butyrylcholinesterase, pseudocholinesterase, K-variant, rivastigmine, BChE, BuChE

## Abstract

Late-onset Alzheimer’s disease (AD) is clinically characterized by a progressive decline of memory and other cognitive functions leading to the loss of the ability to perform everyday activities. Only a few drugs have been approved to treat AD dementia over the past century since the first AD patient was diagnosed. Drugs increasing the availability of neurotransmitters at synapses in the brain are used clinically in the treatment of AD dementia, and cholinesterase inhibitors (ChEIs) are the mainstay of the therapy. A detrimental effect on cognitive function has been reported in patients with pharmacological inhibition of acetylcholinesterase (AChE) by ChEIs and reduced butyrylcholinesterase (BChE) activity due to the single nucleotide polymorphisms. The BChE K-variant (rs1803274), the most common genetic variant of the *BCHE* gene, was thought to reduce enzyme activity reflecting the lower clinical response to rivastigmine in AD patients. During ChEIs therapy, patients carrying reduced-activity BChE do not present such improved attention like patients with the wild-type enzyme. On the other hand, alterations in the *BCHE* gene causing enzyme activity reduction may delay AD onset in patients at risk by preserving the level of cortical acetylcholine (ACh). Based on our previous results, we conclude that SNPs localized outside of the coding sequence, in 5’UTR (rs1126680) and/or intron 2 (rs55781031) of the *BCHE* gene, but not solely K-variant alteration (p.A539T) itself, are responsible for reduced enzyme activity. Therefore, we suspect that not BChE-K itself, but these coexisting SNPs (rs1126680 and rs55781031), could be associated with deleterious changes in cognitive decline in patients treated with ChEIs. Based on the results, we suggest that SNPs (rs1126680) and/or (rs55781031) genotyping should be performed to identify subjects at risk for lowered efficacy ChEIs therapy, and such patients should be treated with a lower rivastigmine dosage. Finally, our sequence analysis of the N-terminal end of N-BChE revealed evolutionarily conserved amino acid residues that can be involved in disulfide bond formation and anchoring of N-BChE in the cell membrane.

## 1. Alzheimer’s Disease (AD)

Alzheimer’s disease (AD), a neurodegenerative disorder characterized by cognitive decline, is the most common form of dementia in elderly individuals. The clinical expression of AD correlates with synaptic damage accompanied by neuronal loss in the brain, particularly in the hippocampus and cerebral cortex [1,2,3,4].

The are many hypotheses regarding the primary cause of AD including cholinergic neuron damage, the accumulation of proteins such as amyloid-β (Aβ) in plaques and hyperphosphorylated-tau in neurofibrillary tangles leading to massive loss of synapses, inflammation, oxidative stress, and others [5,6,7].

By definition, age is the most potent risk factor for late-onset AD [8], followed by the strongest genetic risk factor, the presence of the *APOE*-ε4 allele [9]. Multiple other genetic and environmental factors seem to be involved in late-onset AD pathogenesis. Many studies have shown that an imbalance between the production and clearance of amyloid-β (Aβ) forming amyloid plaques is probably a significant contributor to neurodegeneration and disease development [10,11].

The biochemical investigation of the brains of patients with AD revealed deficits in the enzyme responsible for the levels of ACh. These studies led to the formulation of the cholinergic hypothesis of AD [1,12]. The oldest hypothesis suggests that degeneration of cholinergic neurons in the basal forebrain and the associated loss of cholinergic neurotransmission in the cerebral cortex initiates the progression of cognitive and behavioral dysfunctions seen in patients with AD [13,14,15]. The hypothesis became more convincing when cholinesterase inhibitor therapies were shown to be effective in the improvement of main cognitive functions in patients with AD [16].

The concentration of ACh at synapses of cholinergic neurons is a function of its synthesis and hydrolysis, which is controlled by choline acetyltransferase (Chat) and cholinesterases (ChE), respectively. Mammalian brains contain two sister cholinesterase enzymes: acetylcholinesterase (AChE) and butyrylcholinesterase (BChE). AChE mainly participates in cholinergic neurotransmission by hydrolyzing ACh, but BChE can also hydrolyze it [17]. BChE serves as an endogenous bioscavenger and acts as the first line of defense against toxic compounds, that might inhibit AChE activity [18,19]. Besides, BChE can compensate for AChE in the AChE-knockout mouse [20,21].

In the brain, BChE is found in glia and white matter. Nevertheless, the protein is also associated with neurons, particularly in the hippocampus, amygdala, and thalamus [22,23]. BChE was also found in amyloid plaques and neurofibrillary tangles (NFTs), which suggests that the protein may be involved in the pathogenesis of AD [24,25,26]. Hybrid AChE/BChE tetramers, composed of AChE and BChE dimers, have been found during the development of the brain [27].

## 2. Donepezil and Rivastigmine

Cholinesterase inhibitors are the first-line drugs in the treatment of AD. They inhibit the hydrolysis of acetylcholine by AChE and BChE and, therefore, prolong ACh activity at cholinergic synapses [28,29,30]. Currently, the most prescribed FDA-approved cholinesterase inhibitors for the treatment of AD dementia are donepezil, rivastigmine, and galantamine [31]. All these drugs slow down the symptoms of AD dementia but do not have any effect on neurodegeneration. Donepezil is the most commonly prescribed drug for the treatment of cognitive signs of AD dementia. It is reversible mixed competitive and noncompetitive AChE inhibitor that acts mainly on AChE and shows no significant activity against BChE.

Rivastigmine presents an equal affinity for both AChE and BChE enzymes with a pseudo-irreversible mode of inhibition. It is the only ChEI that produces sustained inhibition without any significant increase in the expression of the target enzymes [32]. Non-carbamylating ChEIs (donepezil, galantamine, and tacrine) increases acetylcholinesterase (AChE) protein expression in patients, whereas the carbamylating agent, rivastigmine, acts with no significant change in AChE protein expression [33,34]. The phenanthrene alkaloid galantamine is a reversible, competitive AChE inhibitor and may act as allosteric modulator of nicotinic acetylcholine receptors. Galantamine, similar to donepezil, shows no significant activity against BChE [35].

## 3. BChE-K Variant

BChE-K substitution (c.1699G>A, p.A539T, rs1803274) is the most common genetic variant detected in the *BCHE* gene. It was presented that K-variant is associated with the lower level of BChE molecules in plasma or with decreased activity caused by the impaired quaternary organization of the tetramer [36]. However, other findings demonstrated that the recombinant K-variant had the same activity and stability as wild-type BChE [37]. We have previously reported that compound SNPs localized outside the coding sequence in 5′UTR and/or intron 2 of the *BCHE* gene, but not solely p.A539T substitution, are responsible for reduced enzyme activity in K allele carriers [38,39].

The BChE-K variant is a long-debated risk factor for AD. It was shown that the K variant was considerably less effective in attenuating the accumulation of Aβ fibrils than BChE wild-type [36,40]. The association between BChE-K variant and AD risk was intensively debated and studied, but no definitive correlation was established since some of the previous results supported the idea [41,42], while other researchers remain doubtful [43,44]. Although many of these studies are based on a meta-analysis, GWAS (genome-wide association study) and cohort study, the results are still inconsistent, and the role of BChE in AD pathology remains unexplained [45,46]. However, our recent study has shown that the BChE-K variant protein with SNP (rs1126680) results in the alteration (p.C-11Y) of 69 amino acid signal peptide, designated N-BChE (the product of K3 haplotype), may play a role in the development of AD [47].

It has been shown that ChEIs therapies have reduced efficacy in dementia patients with BCHE-K variant due to lower enzyme activity. Rivastigmine therapy has been reported to be less effective in AD individuals with *BCHE-K*, especially in the presence of APOE4 [48,49,50]. AD patients with wild-type BChE receiving rivastigmine showed significantly greater treatment responses than patients receiving donepezil. In contrast, BChE K carriers experienced similar effects of therapy with both agents, although adverse events were more frequent in a group of rivastigmine-treated patients.

It was also reported that second ChEI, donepezil can have an adverse effect on cholinergic synapses and therefore on the cognitive function if given to mild cognitive impairment (MCI) patients carrying the K- variant of BChE and particularly among *APOE*-ε4 carriers [51].

It is supposed that pharmacological inhibition of AChE by donepezil and BChE inhibition due to the missense polymorphisms in BChE K-variant carriers leads to an overload of ACh [52], which has a deleterious effect on the cognitive function. On the other hand, alterations in the *BCHE* gene decreasing enzyme activity may delay the onset of disease in patients at risk of AD by preserving the level of cortical ACh.

Studies reporting the influence of BCHE-K genotype on ChEI response are contradictory. Some reports did not find a role of the BChE-K variant on the efficacy of treatment with ChEI [53]. On the other hand, Han et al. concluded that the BChE-K allele was a significant predictor for a poor response to ChEI [50]. We hypothesize that a similar inconsistency was observed in the case of the influence of BCHE-K variant on the enzyme activity. Our recent results have shown that not all BChE-K carriers have lowered enzyme activity, but only individuals with accompanied genetic variants (rs1126680) and/or (rs55781031) designed as the K3 haplotype [39]. Moreover, by analogy, the negative effect of BChE-K carriers on the response to ChEI therapies could only be observed in patients with the K3 haplotype. However, a clinical study should be performed to confirm this hypothesis.

Therefore, we recommend performing molecular testing of *BCHE* SNPs (rs1126680) and/or (rs55781031), instead of BChE-K substitution (rs1803274) in all patients before therapy to identify individuals who are at risk of reduced efficacy of treatment with ChEIs. Moreover, in a group of patients with rs1126680 and rs55781031, the prescription of a lower dosage of rivastigmine or donepezil should be considered to avoid the overload of ACh at synapses [51].

## 4. The N-Terminal Extended BChE Fragment

**Figure 1:**
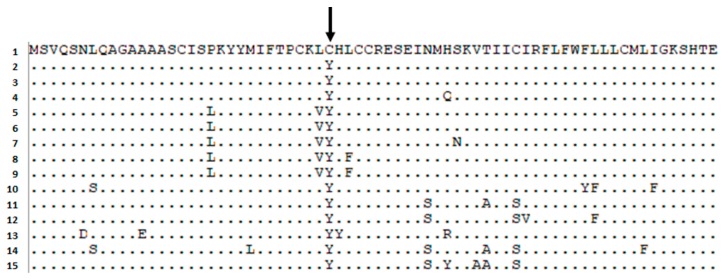
Evolutionary conservation of the N-terminal extended 70 aa. BChE fragment. Position of p.C-11Y (rs1126680) (or p.C31Y counted from the start of the protein) is marked by a black arrow. BLASTP search of BChE proteins of the 14 closest living relatives: 1—(*Homo sapiens*) EAW78592.1; 2—(*Pan troglodytes*) XP_516857.2; 3—(*Pan paniscus*) XP_003817746.1; 4—(*Gorilla gorilla gorilla*) XP_018880258.1; 5—(*Chlorocebus sabaeus*) XP_007970394.1; 6—(*Colobus angolensis palliatus*) XP_011800018.1; 7- (*Piliocolobus tephrosceles*) XP_023069278.1; 8—(*Rhinopithecus bieti*) XP_017745933.1; 9—(*Rhinopithecus roxellana*) XP_010353594.1; 10—(*Nomascus leucogenys*) XP_003256448.1; 11—(*Cebus capucinus imitator*) XP_017358747.1; 12—(*Callithrix jacchus*) XP_002807699.3; 13—(*Pongo abelii*) XP_009237773.2; 14—(*Saimiri boliviensis boliviensis*) XP_003925023.1; 15—(*Aotus nancymaae*) XP_012302784.1.

Intriguingly, amino acid sequence analysis (BLASTP) revealed that N-terminal extended BChE fragment is abundant in mammals. This extended part of BChE is evolutionarily conserved, suggesting that it can play an important role during brain development [27]. Moreover, the only difference between human and chimpanzee at N-terminal BChE end is located at the position rs1126680, resulting in a substitution of cytosine to tyrosine. An analysis of more than 50 BChE sequences of the closest living relatives showed that only human has cysteine at the position of the SNP (rs1126680) (Figure 1). In our study, we have shown that only the N-extended BChE with alteration p.C-11Y is produced in homozygotes. However, it cannot be excluded that also the N-extended wild-type protein may exist in human cells [47].

**Figure 2:**
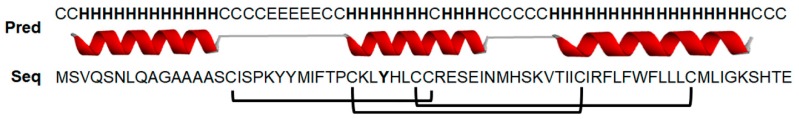
Prediction of the secondary structure of the N-terminal extended 70 aa. BChE fragment with p.C-11Y (rs1126680) (or p.C31Y from the start of the protein) using PSIPRED server [55,56]. H = helix, E = strand, and C = coil. The cysteine residues can form disulfide bridges leading to link and stabilization of 3 helical regions (black parentheses). Disulfide bonds formation between the cysteine residues were predicted using DISULFIND server [57].

A change of tyrosine to cysteine in the protein sequence may introduce potential covalent disulfide bond that could influence the structure of the N-terminal end of BChE. Our secondary structure predictions revealed the presence of three putative alpha-helical regions in the extended N-BChE end that can be linked by three disulfide bonds (Figure 2). Moreover, we assume the N-terminal extension of the BChE may play a role as an anchor that can bind the protein into cellular membranes as it was shown for the N-terminally extended N-AChE [54].
